# Studying of *Trachurus trachurus* (Linnaeus, 1758) Population Reproductive Dynamics in the Eastern Moroccan Mediterranean

**DOI:** 10.1002/ece3.73792

**Published:** 2026-06-04

**Authors:** Hanae Nasri, Khaoula Kasmi, Douaa Slimani, Souad Abdellaoui, Reda Melhaoui, Belkheir Hammouti, Shehdeh Jodeh, Khalid Chaabane, Raed Alkowni

**Affiliations:** ^1^ Laboratory for Agricultural Productions Improvement, Biotechnology and Environment (LAPABE), Faculty of Sciences University Mohammed First Oujda Morocco; ^2^ Euromed University of Fes UEMF Fez Morocco; ^3^ Department of Chemistry An‐Najah National University Nablus Palestine; ^4^ Department of Biology and Biotechnology An‐Najah National University Nablus Palestine

**Keywords:** eastern Morocco, epipelagic fish, gonadal development, reproductive indices, *Trachurus trachurus*

## Abstract

Horse mackerel ( *Trachurus trachurus* , Linnaeus, 1758) is an epipelagic fish commonly found in Moroccan Mediterranean waters. This study was aimed at studying the ecology by monitoring gonadal development and reproductive indices of *T. trachurus* populations along the eastern Moroccan Mediterranean coast over a 12‐month period. For that, a total of 390 specimens were sampled, with total lengths ranging from 7.8 to 33.8 cm and weights from 3.78 to 310.52 g. Several reproductive indices were analyzed. Monthly monitoring of the gonado‐somatic index (GSI) identified two spawning periods for *T. trachurus* : the first in late winter‐early spring (February–April), and the second in summer (July–September), confirming that this species is a fractional spawner fish. The hepato‐somatic index (HSI) followed a similar trend to GSI, reflecting the energy allocation for reproduction. The size at first sexual maturity (L50) was estimated at 23.5 cm for females and 22.5 cm for males, representing critical metrics for fisheries management, as they help set regulatory guidelines, such as minimum landing sizes, and are also a prerequisite for estimating spawning stock biomass. The overall sex ratio showed a predominance in favor of males (62%) over females (38%). These results represent a valuable contribution to a better understanding of the biology of *T. trachurus* , and to the implementation of stock assessment to ensure the sustainability of catches.

## Graphical abstract

Horse mackerel ( *Trachurus trachurus* ) populations along the eastern Moroccan Mediterranean coast were studied over 12 months using 390 specimens to assess reproductive ecology. The species showed two spawning periods—late winter to early spring and summer—and exhibited fractional spawning, with males being more predominant. Size at first maturity was about 23.5 cm for females and 22.5 cm for males, and reproductive indices such as GSI and HSI highlighted clear patterns of energy allocation for reproduction.

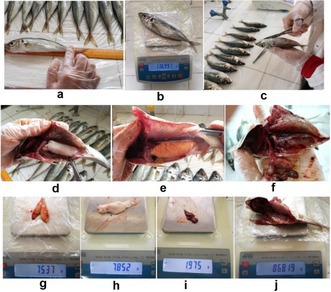

## 1 Introduction

Pelagic fish are species that live between the surface and the bottom of the oceans and are typically found in the continental shelf waters (Petrik et al. 2019). They include several hundred species with common characteristics: a dark blue coloration on the back and silver on the belly to protect them from predators, an elongated shape, and an often‐gregarious lifestyle. Horse mackerel ( *Trachurus trachurus* ), sardines ( *Sardina pilchardus* ), anchovies ( *Engraulis encrasicolus* ), herring ( *Clupea harengus* ), round sardinella ( *Sardinella aurita* ), and flat sardinella ( *Sardinella maderensis* ) (Alheit and Peck 2019; Hunnam 2021; Nasri et al. 2021) are the main species of small pelagic fish in the Mediterranean. These species account for significant catch volumes worldwide and are also the most sought‐after by the fish processing industry for the production of fishmeal and fish oil (FAO 2022).

Morocco's geographical position provides it with two coastlines, granting access to large fish stocks. Morocco's Atlantic coastline is known for its wealth of fish resources, thanks to the upwelling phenomenon. The Mediterranean coast also plays a role in the catch of small pelagic fish. According to the most recent stock assessment report published in 2023 by the Moroccan National Federation of Fish Processing and Valorization Industries (FENIP), total catches of this category of fish resources were estimated at approximately 1.5 million tons in 2022 (FENIP 2023).

*Trachurus trachurus* is widely distributed in this area, and is of major economic importance in occupying an important place in landings. This species ranks third in total small pelagic catch, after sardines and mackerel (INRH 2019). Due to its affordable price and year‐round availability, this fish is in high demand by the Moroccan population. Its flesh is highly appreciated by consumers both fresh and canned due to its high nutritional value particularly its richness in polyunsaturated fatty acids (Aiyeloja et al. 2022). As a result, horse mackerel is subject to fishing pressure from both seiners and trawlers and requires careful monitoring and further scientific study to better understand its population dynamics, control its life cycle and assess its stock. Population density in *T. trachurus* can fluctuate across space and seasons, largely depending on environmental conditions and food availability, since the species forms large schools and shows seasonal movements. It mainly inhabits pelagic and neritic waters of the continental shelf, where habitat characteristics such as water temperature, depth, and productivity strongly influence its distribution. In addition, *T. trachurus* plays a central role in marine food webs as both a predator and prey: it feeds mainly on zooplankton and small fish, while being consumed by larger predatory fish, marine mammals, and seabirds, indicating a potentially strong predation pressure. Finally, because it relies on similar prey resources as other small pelagic species (e.g., sardines and anchovies), it is likely affected by interspecific competition, which may shape its feeding success and spatial distribution.

Climate change is causing a progressive increase in ocean temperatures and a greater occurrence of intense marine heatwave events worldwide. These environmental changes may strongly influence fish reproductive processes and affect their reproductive success, in some cases, even induce sex reversals or shifts in geographical range. The most observed type of temperature‐dependent sex determination in fishes is gonadal masculinization at elevated temperatures (Lema et al. 2024). The reproduction of Horse mackerel is a complex process (Alvarez et al. 2023; Nasri et al. 2024). It is under endocrine control (hormone regulation), and is highly influenced by environmental conditions, particularly, temperature and photoperiod, which affect gonad maturation, the development of secondary sexual characteristics and reproductive behavior. These changes lead to a breeding period when environmental conditions are optimal for development (Waldron and Kerstan 2001). The spawning period varies by region due to key factors such as latitude and temperature (Ferreri et al. 2019).

The “Eastern Moroccan Mediterranean” refers to Morocco's northeastern Mediterranean coastline, which is known for its beautiful beaches like Saïdia and the cities of Nador and Oujda. The eastern Moroccan Mediterranean coast is a fish‐rich area, with three landing ports (Al Hoceima, Beni Nsar and Ras kebdana) and is characterized by a high concentration of fishing activity, which represents a very important economic interest. Although the eastern Mediterranean is rich in pelagic fish resources, relatively few studies have investigated the reproductive biology of *T. trachurus* , highlighting the importance of the present study. In this context, this study aimed to investigate the reproductive biology of this species, particularly their reproductive activity in pelagic and demersal fishery catches along the eastern Moroccan Mediterranean coast to increase the knowledge of their resilience toward the current global climate changes. This study investigated their reproductivity based on key parameters as sexual maturity, gonado‐somatic index (GSI), hepato‐somatic index (HSI), sex ratio, and size at first sexual maturity.

## 2 Materials and Methods

### 2.1 Site Study and Sample Collection

The specimens were collected in the northeastern Moroccan Mediterranean, particularly in the areas of Al Hoceima, Beni Nsar, and Ras Kebdana. The species is mainly caught by commercial fishermen using bottom trawlers, at depths ranging from 50 to 510 m. The samples were then transported to the wholesale market of the National Office of Fisheries (ONP) in Oujda, where the fish were landed in the early morning hours.

Monthly sampling campaigns were conducted at the wholesale market, during which 30 to 40 fish were processed per sampling session. Samples were taken randomly from October 2017 to September 2018 to ensure a representative sample. Once the specimens were collected, they were transported in a cool box to the Laboratory of Agricultural Production Improvement, Biotechnology and Environment, so that any fish still fresh could be processed quickly. A total of 390 *T. trachurus* specimens were collected (131 males, 79 females and 180 undetermined) on a regular basis to obtain a complete reproductive cycle for this species (Table 1). The high proportion of undetermined individuals was mainly associated with immature specimens presenting poorly differentiated gonads, which made macroscopic sex identification difficult. These individuals were therefore excluded from analyses requiring sex determination in order to avoid potential misclassification and ensure the reliability of the reproductive parameters estimated in this study. Despite the relatively high number of undetermined specimens, the overall patterns observed, particularly regarding spawning periods, sex ratio trends, gonado‐somatic index variation, and size at first sexual maturity, remain consistent with results previously reported in the literature for *T. trachurus* in other Mediterranean and Atlantic regions. This consistency supports the robustness and reliability of our findings despite the limitations associated with macroscopic identification of immature individuals.

**TABLE 1:** Monthly variation in the number of male, female, and undetermined *Trachurus. trachurus* specimens collected during the study period.

	Males	Females	Undetermined
October	0	0	28
November	0	1	28
December	0	0	25
January	0	0	25
February	21	8	0
March	25	18	0
April	11	3	15
May	3	9	20
June	0	0	30
July	26	11	0
August	25	18	4
September	20	11	5
	131	79	180

### 2.2 Measuring and Weighing

**FIGURE 1:**
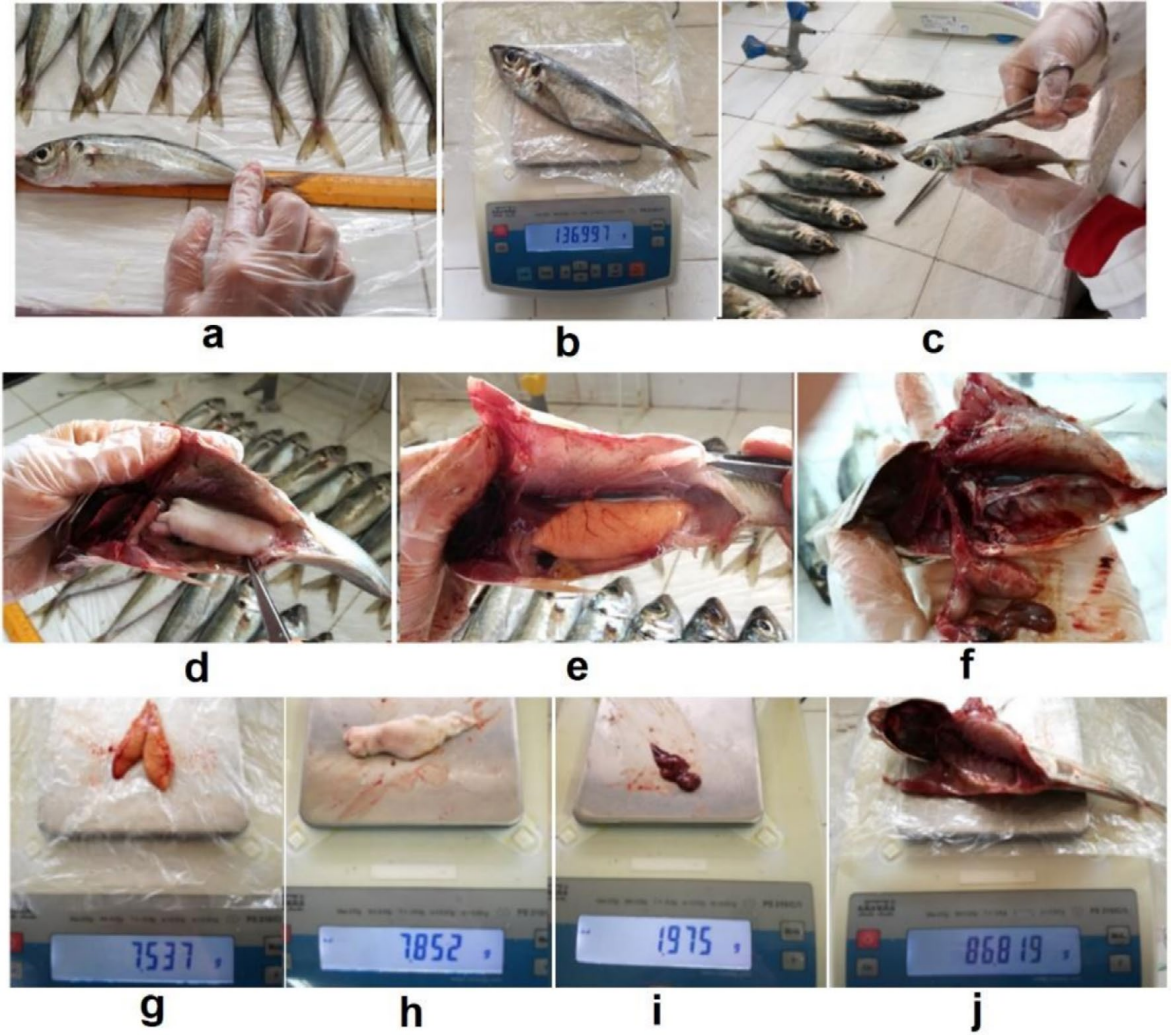
Simultaneous measurements and weighing with dissection; (a) Total length (Lt) of the fish, (b) Total weight of the fish, (c) Incision of the abdominal cavity for sex determination, (d) Male gonads in place within the abdomen, (e) Female gonads in place within the abdomen, (f) Organ sampling, (g) Weight of female gonads, (h) Weight of male gonads, (i) Liver weight, (j) Eviscerated weight of the fish.

Length measurements were taken using a graduated ichthyometer (±0.1 mm), and various weights (±0.01 g) were recorded including total weight, eviscerated weight, liver weight and gonad weight (Figure 1).

### 2.3 Sex Determination

**FIGURE 2:**
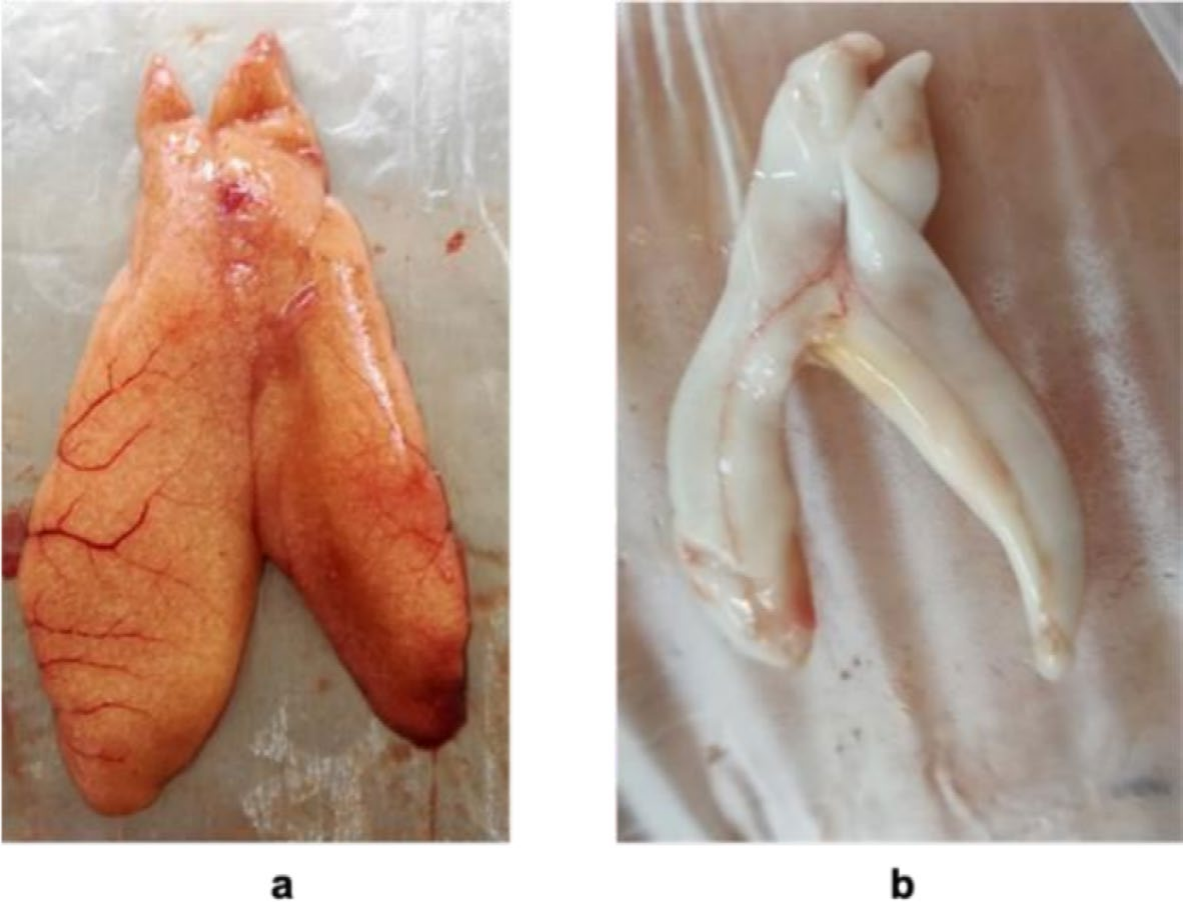
Male and female reproductive system of *Trachurus trachurus* ; (a) Female gonads, (b) Male gonads (Photo: H. Nasri 2018).

Due to the absence of sexual dimorphism (apparent distinctive features) in this species, sex determination requires an incision of the fish's abdomen from the anus to the operculum. In mature specimens, sex differentiation is based on observation of the gonads with the naked eye. The ovaries are typically tubular and granular, while the testes are flat, white, and often display a wavy line along the ventral margin (Figure 2).

### 2.4 Sex Ratio Determination

The sex ratio (SR) generally reflects the rate of masculinity or femininity of the population under consideration. The numerical proportions of the sexes are expressed as percentages. It can be expressed and calculated in different ways (Gherram et al. 2018). Overall sex ratio is defined as the proportion of male individuals in relation to the number of females.

### 2.5 Determining the Spawning Period

To determine the spawning period, we analyzed the gonado‐somatic index (GSI) and the hepato‐somatic index (HSI). GSI represents the ratio of gonad weight to eviscerated body weight, and is calculated as follows:

$$\mathrm{GSI}=\left(\mathrm{Wg}/\text{We}\right)\times 100$$

where Wg is gonad weight (g) and We is eviscerated body weight (g).

The HSI represents the ratio of liver weight to eviscerated body weight and is calculated as follows:

$$\mathrm{HSI}=\left(\mathrm{Wf}/\text{We}\right)\times 100$$

where Wf is liver weight (g) and We is eviscerated body weight (g).

GSI and HSI data for *T. trachurus* are processed based on size, sex, and month. These data are then plotted on graphs to follow the temporal evolution of GSI and HSI throughout the year, with the aim of determining the spawning period and the origin of the reserves used during the species' spawning season.

### 2.6 Study of Sexual Maturity

Determination of sexual maturity stages of *T. trachurus* is based on macroscopic observation of the male and female gonads using morphological criteria.

This macroscopic assessment (Table 2) provides a more reliable and precise classification of maturity stages of gonad development based on well‐defined criteria (Fontana 1969; Holden and Raitt 1974; ICES 2008).

**TABLE 2:** Macroscopic stages of *Trachurus trachurus* ovaries.

Stage	Females (Ovaries)	Males (Testes)
I—Immature	Fine, translucent to pink ovary, oocytes invisible	Small, translucent, very thin testis
II—Developing immature/sexually resting adults	Ovary not bulky, pinkish, intense vascularization in resting fish, oocytes invisible	Whitish, more or less symmetrical testis
III—Beginning of maturation	Medium‐sized ovary, pale pink to light orange, some oocytes sometimes visible	Wider, firm, white testis, no liquid flows if incised
IV—Spawning/sperm emission	Very large ovary filling abdominal cavity, thin transparent wall, hyaline oocytes visible and expelled under slight pressure	Very large, soft testis, sperm flows under abdominal pressure
V—Post‐spawning/post‐emission	Flaccid, highly vascularized ovary, red color, thick ovarian wall	Large, very flaccid, highly vascularized testis, no sperm released under pressure

### 2.7 Size at First Sexual Maturity (L50)

A logistic function was used to relate the proportions of mature individuals to the total length of the fish, as it provides a better statistical interpretation and is closer to the actual values observed during sampling (Taieb et al. 2010):

$$\mathrm{Pr}=1/\left[1+\exp-a\left(L-L_{50}\right)\right]$$

where Pr is proportion of mature individuals, *a* is slope, *L* is total length in cm, and *L*_50_ is size at first sexual maturity.

## 3 Results and Discussion

**FIGURE 3:**
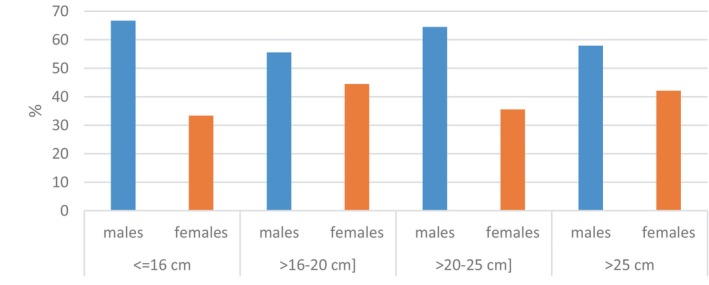
Sex distribution of *Trachurus trachurus* by size class.

Biological data on the reproduction of *T. trachurus* , collected over a period of one year in the waters of the eastern Mediterranean of Morocco, were analyzed to enhance knowledge of the species reproduction for stock management purposes. Male lengths ranged from 13 to 33.4 cm, while female lengths ranged from 12.5 to 33.8 cm. Female weights ranged from 17.4 to 310.52 g, and male weights from 19.32 to 292.69 g. The numerical distribution of sexes or sex ratio is an important biological index, as the proportion of males and females can influence the reproductive success of the species. The sex ratio is an essential parameter for maintaining a balance between the sexes within a population (Argasinski 2012). The overall sex ratio (SR) is 1.64 (SR = males/females). The masculinity rate is 62.07%, against a femininity rate of 37.93%. According to the Chi‐square (*χ* ^2^) test at a 95% threshold, the overall sex ratio compared to a balanced sex ratio (one male for one female) shows a significant difference between males and females at the 5% threshold (*p* < 0.05). This index was analyzed as a function of size (Figure 3), to track the dynamics of the proportion of both sexes and their distribution. Changes in sex ratio as a function of size can be used to reconstruct the size structure of commercial catches by sex and can also provide information for studying growth (Bensahla Talet 2014).

Our observations indicate a numerical dominance of males. These results are similar to previous studies on horse mackerel in the Mediterranean (Gherram 2019; Rahmani 2020). The variation in this index could be attributed to the lifestyle of *T. trachurus* , which is a gregarious bentho‐pelagic fish. It is therefore possible that certain schools of horse mackerel are predominantly male or female (Carbonara et al. 2012; Wahbi et al. 2015). Fluctuations in the sex ratio are thought to be caused by behavioral phenomena (segregation of populations, etc.) which lead to dispersion and segregation of the sexes. The difficulty of interpreting fluctuations in this ratio is due to several factors, such as the behavior of the species, the spawning and mortality period, sampling methods, and the aggregation of individuals of the same sex (Chauvet 1986). Ward et al. (2020) discuss social aggregation as a widespread and important phenomenon in fishes. The authors emphasize that group‐living fishes do not always form schools randomly. Rather, schools may be structured according to several criteria, including species, size, age, behavior, familiarity, kinship, and sex. In this context, sexually biased schools should not be considered as an observation specific to *T*. *trachurus*, but rather as part of a broader phenomenon observed in gregarious fishes. The sexual composition of a school may result from mechanisms of social recognition, selective social attraction, and behavioral segregation. These mechanisms may influence association preferences and, ultimately, the composition of fish groups.

In addition, a recent study on horse mackerel from the Western Mediterranean Sea reported interannual variation in the sex ratio between 2012 and 2020. For example, the female‐to‐male ratio was 2.6:1 in 2017, whereas it was 0.4:1 in 2020 (Rodríguez‐Castañeda et al. 2024). These findings suggest that males and females may not be evenly distributed in catches, possibly reflecting temporal variation, reproductive dynamics, spatial segregation, or sampling effects.

### 3.1 Monthly Variation of Sexual Maturity

**FIGURE 4:**
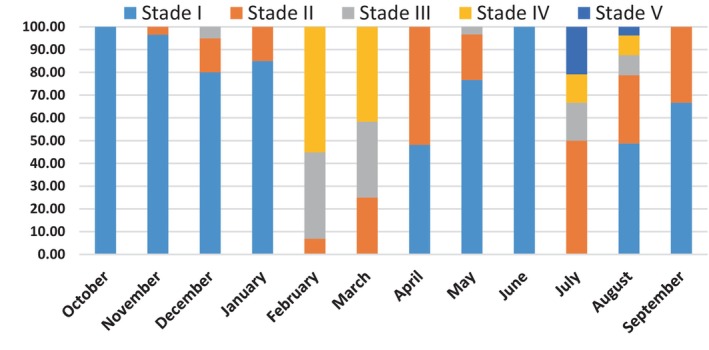
Monthly evolution of the percentages of sexual maturity stages in *Trachurus trachurus* observed at the macroscopic scale.

The monthly variation in the percentage of sexual maturity stages shows that horse mackerel are at different stages of sexual maturity throughout the year, with varying proportions (Figure 4).

The highest percentages of sexually mature, spawning, and post‐spawning individuals (stages III, IV, and V) are observed during two distinct periods, the first from February to March and the second from July to August. Immature or sexually resting individuals (stages I and II) are present year‐round, but the highest proportions occur in autumn and spring. During these two seasons, most individuals have completed breeding and are entering their sexual resting period. These results suggest that horse mackerel undergo two main breeding periods per year, the first in late winter to early spring and the second in summer. However, some individuals may still reproduce in December and May.

### 3.2 Gonado‐Somatic Index (GSI)

The assessment of gonad weight variation over the 12‐month study period enables the identification of the reproductive season of a given species. Average GSI values for female *T. trachurus* calculated for each month are shown in Figure 4. The temporal variation of the gonado‐somatic index shows the presence of two periods of sexual maturation of the gonads, characterized by a regular increase in GSI starting in February and continuing until March, followed by a second, less pronounced rise between July and August. These two phases of GSI increase correspond to gonad maturation. This is followed by two spawning phases, the first from March to April and the second from August to September, corresponding to a significant decrease in GSI. Additionally, a slight increase in GSI is observed in May, but it remains negligible compared to the two peaks, winter‐early spring and summer. After the spawning periods, the species enters a sexual resting phase from September to January.

The temporal variation of the gonado‐somatic index of females reveals two main peaks: with a main peak in March (8.92 ± 1.2) and a secondary peak in August (2.71 ± 0.82). The decrease in GSI following these peaks is due to the release of ova during spawning and the regression of the ovary after egg deposition. Monthly monitoring of this parameter allows us to conclude two reproductive periods in females. The first, and most important, occurs between February and April, while the second, less pronounced, between July and September.

**FIGURE 5:**
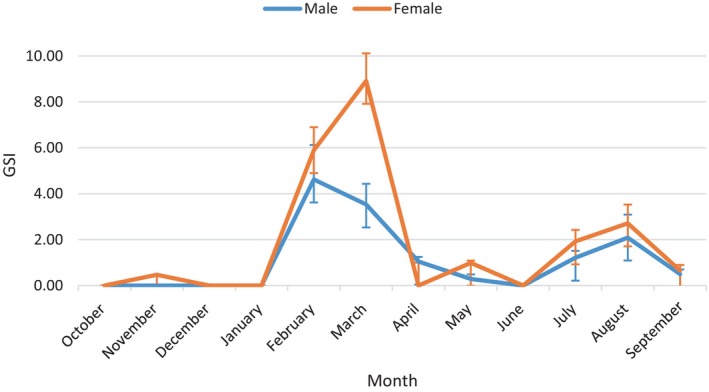
Monthly evolution of the GSI in males and females of *Trachurus trachurus* along the eastern coast of the Moroccan Mediterranean.

The evolution of GSI in males highlights the process of gonad maturation with a first period between February and March, and a second period between July and August. Sperm emission also follows two periods, the first in early spring (March and April) and the second in summer (between August and September). This is followed by the sexual rest phase, marked by minimum GSI values recorded from September to January (Figure 5). Male GSI follows a cyclical pattern identical to that of females. Monthly monitoring of this parameter confirms the two breeding periods for males. The first, most significant, occurs between February and April, and the second, of less importance, takes place between July and September.

The monthly evolution of GSI follows a similar pattern for both sexes, with two distinct peaks. This suggests that *T. trachurus* is a fractional‐spawning fish, where oocytes do not develop simultaneously but are released in several successive waves. In fact, gonads are characterized by asynchronous oocyte development, with multiple batches of oocytes at various stages of development present at the same time, without any particular class predominating. These results are in line with the findings of Abaunza et al. (2003) and Murua and Saborido‐Rey (2003).

The GSI percentages in females are higher than those in males due to the larger size of the ovaries. The gonado‐somatic index is considered the main index of gonadal maturation. Its increase indicates gametogenesis, while its decrease indicates active spawning. The results show a spatio‐temporal parallelism in the gonad maturation cycle and good synchronization of the reproductive peak in both sexes. GSI monitoring allowed us to identify two breeding periods for *T. trachurus* : one in late winter/early spring between February and April, and the second in summer between July and September.

Several studies carried out in the Mediterranean on the reproductive cycle of the *T. trachurus* using both macroscopic and histological criteria have found that the breeding period varies across regions (Table 3).

**TABLE 3:** Reproduction period of the *Trachurus trachurus* according to the area.

Area	Period	References
Algeria	February to July	Rahmani (2020)
Algeria	October to March	Tahari (2011)
Algeria	January to May	Gherram et al. (2018)
Northern Aegean Sea	April to August	Aydin and Erdoğan (2018)
Gulf of Skikda, Algeria	December and April	Azzouz et al. (2019)
Northwestern Mediterranean	Winter–Spring	Raya and Sabatés (2015)
Eastern Adriatic	Late winter and early spring	Jardas et al. (2004)

### 3.3 Hepato‐Somatic Index (HSI)

**FIGURE 6:**
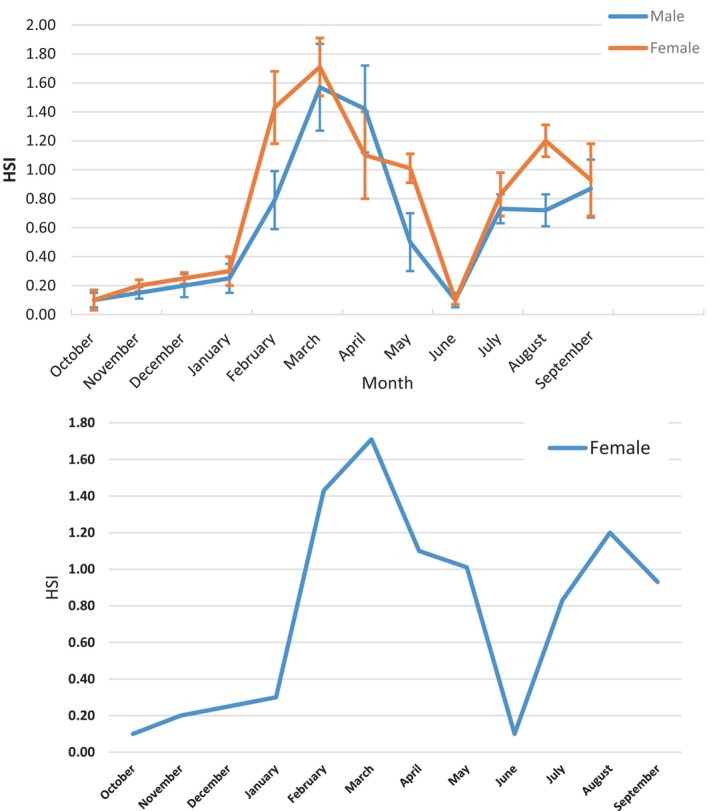
Monthly evolution of the HSI in females and males of *Trachurus trachurus* along the eastern coast of the Moroccan Mediterranean.

The HSI data obtained are plotted in Figure 5. In females, monthly HSI monitoring revealed two peaks: a primary peak in March reaching 1.71 and a secondary peak in August equal to 1.2. Monthly HSI monitoring in males reveals a cyclical pattern identical to that of females (Figure 6).

**FIGURE 7:**
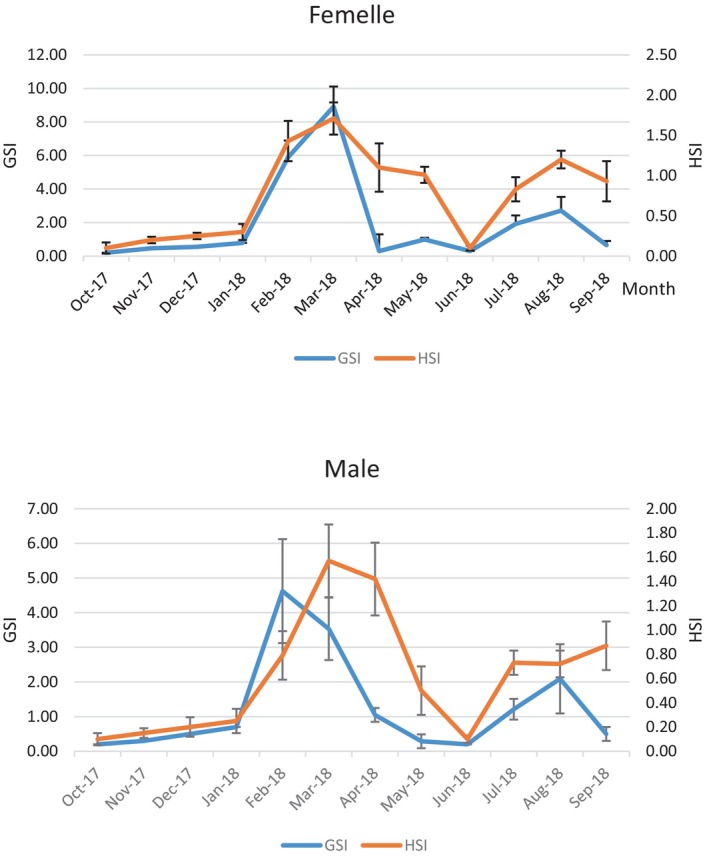
Monthly evolution of GSI and HSI in male and female *Trachurus trachurus* along the eastern coast of the Moroccan Mediterranean.

The peak HSI recorded in males in March, at 1.57, corresponds to a GSI of 3.53, with a secondary peak in September at 0.87. The results obtained show a similar trend *between* the hepato‐somatic and gonado‐somatic indexes. In both males and females, monthly variations in HSI generally follow a pattern similar to that of GSI (Figure 7).

In other words, an individual with relatively heavy ovaries or testes has a high chance of also having a relatively heavy liver. This suggests that gonad maturation is not preceded by lipid accumulation in the liver. Therefore, our findings suggest that *T. trachurus* may be considered a fatty fish because its lipid reserves appear to be mainly stored in body tissues rather than being exclusively accumulated in the liver. Seasonal studies showed that non‐polar lipids, mainly triacylglycerols, were the dominant lipid fraction, indicating the presence of important storage lipids (Bandarra et al. 2001). Moreover, the positive relationship between gonad weight and liver weight suggests that gonadal maturation is not preceded by liver lipid depletion. This agrees with Azzouz et al. (2019), who found similar seasonal patterns between HSI and GSI and concluded that the energetic reserves used for gonadal development are mainly derived from muscular or mesenteric lipid reserves. Ndjaula et al. (2009) and Van Damme et al. (2014) also showed that GSI, HSI, condition factor, and lipid content increased during oocyte development or spawning, supporting the view that reproduction in *T. trachurus* is linked to the general energetic condition of the fish rather than to the mobilization of liver reserves alone.

### 3.4 Size at First Sexual Maturity

**FIGURE 8:**
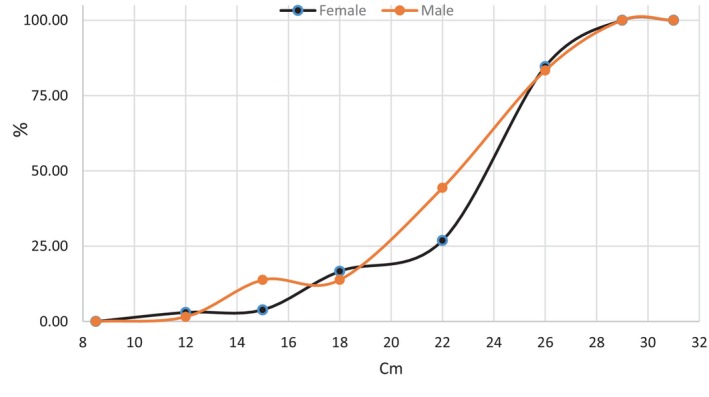
Sexual maturity of female and male *Trachurus trachurus* along the eastern coast of the Moroccan Mediterranean.

Not all individuals in the population reach sexual maturity at the same time. Variations in the percentages of mature individuals by sex and size class in *T. trachurus* are shown in Figure 8. The size at which 50% of individuals are mature (L50) was estimated at a total length of 23.5 cm for females and 22.5 cm for males. The size at which 100% of individuals are mature is reached at 27 cm for both sexes. The lengths corresponding to 25% and 75% maturity are respectively L25 = 19.3 cm and L75 = 25.1 cm for males and L25 = 21.6 cm and L75 = 25.2 cm for females. These results show that males reach sexual maturity at a smaller size than females.

These values were higher than those reported by Carbonara et al. (2012) and Azzouz et al. (2019) who found 14, 18.42, and 18.9 cm, respectively. These differences may be attributed to geographical variability, stock structure, environmental conditions, fishing pressure, sampling design, as well as methodological differences in the assessment of gonadal maturity stages. Previous studies have shown that the length at first maturity in *T. trachurus* can vary considerably among regions. In addition, macroscopic assessment of gonadal maturity may lead to misclassification, particularly between immature individuals and those in resting or post‐spawning stages, which may affect the estimation of the maturity ogive. Our findings are consistent with those reported by El Achi et al. (2021) along the North Atlantic Moroccan coast, whose results closely agree with those obtained in the present study. These authors estimated the length at first sexual maturity at 22.75 cm for females and 21.75 cm for males, values that are comparable to our estimates of 23.5 cm for females and 22.5 cm for males. Moreover, the comparative table presented in the same study shows that the L50 of *T. trachurus* varies markedly among regions. For instance, it is approximately 19 cm for coastal individuals and 23 cm for offshore individuals in the Bay of Biscay, while it may reach 26.25 cm in females from British waters. These findings confirm that the length at first maturity is not fixed in this species, but is likely influenced by geographical area, environmental conditions, stock structure and fishing pressure.

In a population, knowledge of the size of first sexual maturity is crucial for stock preservation. It provides information on the proportion of the stock capable of ensuring species renewal and serves as a reference for the minimum legal catch size. The size at first sexual maturity is therefore a key parameter in the rational management of stocks (Nunes et al. 2020), aiming to prohibit the capture of fish that have not yet reached this size, and allowing them the opportunity to spawn at least once in their lifetime. This is only possible by fixing the mesh size of fishing nets (Ghorbel et al. 2002). The aim is not then to protect immature fish in principle, but to ensure sufficient fecundity for stock regeneration.

The results of this study provide important insights into the reproductive ecology of *T. trachurus* along the eastern Moroccan Mediterranean coast, highlighting clear temporal patterns in gonadal development and reproductive activity across the annual cycle. The observed seasonal fluctuations in gonadosomatic indices and maturity stages are consistent with earlier findings from other regions of the species' distribution, which similarly report a pronounced spawning peak during warmer months driven by favorable environmental conditions (Abaunza et al. 2008). Such patterns underscore the strong influence of temperature, food availability, and regional hydrodynamics on reproductive timing and success in small pelagic fishes. Moreover, the continuous monthly monitoring employed in this study strengthens the reliability of the reproductive indices, offering a valuable baseline for future stock assessments and conservation planning. Given increasing anthropogenic pressures and environmental variability in the Mediterranean Sea, understanding the reproductive dynamics of commercially important species like *T. trachurus* is essential for developing adaptive management strategies that ensure stock sustainability and ecosystem stability.

## 4 Conclusion

The results of our study on the reproduction of *T. trachurus* along the eastern coast of the Moroccan Mediterranean serve as a valuable database that complements existing research on this species throughout the Mediterranean. By monitoring the GSI and HSI indices, combined with macroscopic observation, we were able to identify the breeding period of *T. trachurus* .

Two main breeding periods were identified in *T. trachurus* , with peak gamete production occurring in late winter‐early spring and summer. The exploitable stock contains a higher proportion of males than females. The size of first sexual maturity (L50) differs between sexes, estimated at 23.5 cm for females and 22.5 cm for males, indicating that males mature earlier. Our analysis suggests that over 70% of sampled *T. trachurus* have not yet reached this size. It is therefore vital to preserve this resource through sustainable stock management.

This study has identified key aspects of *T. trachurus* reproduction. The estimated spawning indices provide essential data for stock assessment, contributing to improved management of the exploitable stock of this species in the Moroccan Mediterranean.

## Author Contributions

**Hanae Nasri:** conceptualization (equal), visualization (equal). **Khaoula Kasmi:** conceptualization (equal). **Douaa Slimani:** methodology (equal). **Souad Abdellaoui:** methodology (equal). **Reda Melhaoui:** software (equal). **Belkheir Hammouti:** writing – original draft (equal). **Shehdeh Jodeh:** writing – original draft (equal). **Khalid Chaabane:** formal analysis (equal). **Raed Alkowni:** validation (equal), writing – review and editing (equal).

## Funding

The authors have nothing to report.

## Conflicts of Interest

The authors declare no conflicts of interest.

## Data Availability

The data presented in this study are available at this link: https://drive.google.com/file/d/1f3U09ul_RF5CKll4j2bG02o0aDswy9XV/view?usp=sharing.

## References

[ece373792-bib-0001] Abaunza, P. , L. Gordo , C. Karlou‐Riga , et al. 2003. “Growth and Reproduction of Horse Mackerel, *Trachurus trachurus* (*Carangidae*).” Reviews in Fish Biology and Fisheries 13: 27–61. 10.1023/A:1026334532390.

[ece373792-bib-0002] Abaunza, P. , A. G. Murta , N. Campbell , et al. 2008. “Stock Identity of Horse Mackerel ( *Trachurus trachurus* ) in the Northeast Atlantic and Mediterranean Sea: Integrating the Results From Different Stock Identification Approaches.” Fisheries Research 89, no. 2: 196–209.

[ece373792-bib-0003] Aiyeloja, J. O. , S. N. Deekae , O. M. G. Abu , and O. A. Akinrotimi . 2022. “Fatty Acid Profile of Atlantic Horse Mackerel ( *Trachurus trachurus* ) Oil Obtained Using Different Extraction Methods.” Asian Journal of Fisheries and Aquatic Research 17: 1–8. 10.9734/ajfar/2022/v16i730392.

[ece373792-bib-0004] Alheit, J. , and M. A. Peck . 2019. “Drivers of Dynamics of Small Pelagic Fish Resources: Biology, Management and Human Factors.” Marine Ecology Progress Series 617–618: 1–6. 10.3354/meps12985.

[ece373792-bib-0005] Alvarez, P. , M. M. Angélico , E. Blom , et al. 2023. “Sharing Experiences on Conducting Two Egg Production Methods During the International Mackerel and Horse Mackerel Egg Surveys (MEGS).” In ICES Annual Science Conference.

[ece373792-bib-0006] Argasinski, K. 2012. “The Dynamics of Sex Ratio Evolution Dynamics of Global Population Parameters.” Journal of Theoretical Biology 309: 134–146. 10.1016/j.jtbi.2012.05.025. 22683379

[ece373792-bib-0007] Aydin, G. U. , and Z. Erdoğan . 2018. “Some Reproductive Characteristics of *Trachurus trachurus* , (Linneaus, 1758) From Edremit Bay (Northern Aegean Sea, Turkey).” Balıkesir Üniversitesi Fen Bilimleri Enstitüsü Dergisi 20: 164–176. 10.25092/baunfbed.412525.

[ece373792-bib-0008] Azzouz, S. , L. Mezedjri , and A. Tahar . 2019. “Reproductive Cycle of the Pelagic Fish Saurel *Trachurus trachurus* (Linnaeus, 1758) (*Perciformes carangidae*) Caught in the Gulf of Skikda (Algerian East Coast).” Biodivers Journal 10: 13–20. 10.31396/Biodiv.Jour.2019.10.1.13.20.

[ece373792-bib-0009] Bandarra, N. M. , I. Batista , M. L. Nunes , and J. M. Empis . 2001. “Seasonal Variation in the Chemical Composition of Horse‐Mackerel ( *Trachurus trachurus* ).” European Food Research and Technology 212, no. 5: 535–539. 10.1007/s002170100299.

[ece373792-bib-0010] Bensahla Talet, L. 2014. Biologie et Dynamique de la Population du Pageot Argenté *Pagellus acarne* (Risso, 1827) Pêché Dans la Baie d'Oran. PhD Thesis. University of Oran1‐Algeria.

[ece373792-bib-0011] Carbonara, P. , L. Casciaro , I. Bitetto , and M. T. Spedicato . 2012. “Reproductive Cycle and Length at First Maturity of *Trachurus trachurus* in the Central‐Western Mediterranean Seas.” Biologia Marina Mediterranea 19: 204. 10.31396/Biodiv.Jour.2020.11.2.389.39.

[ece373792-bib-0012] Chauvet, C. 1986. Exploitation des Poissons en Milieu Lagunaire Méditerranéen: Dynamique du Peuplement Ichtyologique de la Lagune de Tunis et des Populations Exploitées par des Bordigues (Muges, Loups, Daurades). PhD Thesis. ANRT.

[ece373792-bib-0013] El Achi, A. , M. Nafia , K. Manchih , A. Baali , and M. Moncef . 2021. “Reproductive Biology of Horse Mackerel *Trachurus trachurus* (Linnaeus, 1758) in the North Atlantic Moroccan Coast.” Egyptian Journal of Aquatic Biology and Fisheries 25, no. 3: 647–666. 10.21608/ejabf.2021.179981.

[ece373792-bib-0014] FAO . 2022. The State of World Fisheries and Aquaculture 2022, Towards Blue Transformation. FAO.

[ece373792-bib-0015] FENIP . 2023. Statistical Report FENIP 2022–2023 (October Edition). National Federation of Fish Processing and Valorization Industries. FICHE STATISTIQUE FENIP 2022–2023.

[ece373792-bib-0016] Ferreri, R. , R. S. McBride , M. Barra , et al. 2019. “Variation in Size at Maturity by Horse Mackerel ( *Trachurus trachurus* ) Within the Central Mediterranean Sea: Implications for Investigating Drivers of Local Productivity and Applications for Resource Assessments.” Fisheries Research 211: 291–299. 10.1016/j.fishres.2018.11.026.

[ece373792-bib-0017] Fontana, A. 1969. Etude de la Maturité Sexuelle des Sardinelles Sardinella Eba (Val) et Sardinella Aurita C. et V. de la Région de Pointe‐Noire. Cah. O.R.S.T.O.M., VII.

[ece373792-bib-0018] Gherram, M. 2019. Ecobiologie de Trois Taxons de Saurel, *Trachurus trachurus* (L, 1758), *Trachurus mediterraneus* (S, 1868) et *Trachurus picturatus* (B, 1825) de la baie d'Oran: Dynamique de Population et Diversité Génétique. PhD Thesis. University of Oran, Algeria.

[ece373792-bib-0019] Gherram, M. , A. B. Talet , F. Dalouche , and S. M. E. A. A. Ayad . 2018. “Study of Reproductive Aspects of *Trachurus trachurus* (Linnaeus, 1758) From Western Coast of Algeria.” Indian Journal of Geo‐Marine Sciences 47: 2469–2476.

[ece373792-bib-0020] Ghorbel, A. O. , M. N. Bradai , and A. Bouain . 2002. “Période de Reproduction et Maturité Sexuelle de Symphodus (Crenilabrus) tinca (Labridae), des côtes de Sfax (Tunisie).” Cybium 26: 89–92.

[ece373792-bib-0021] Holden, M. J. , and D. F. S. Raitt . 1974. Manual of Fisheries Science. Part 2‐Methods of Resource Investigation et Their Application. Doc. Tech. FAO Sur Peches FAO‐Doc. Tec. FAO Sobre Pesca FAO.

[ece373792-bib-0022] Hunnam, K. 2021. “The Biology and Ecology of Tropical Marine Sardines and Herrings in Indo‐West Pacific Fisheries: A Review.” Reviews in Fish Biology and Fisheries 31: 449–484. 10.1007/s11160-021-09649-9.

[ece373792-bib-0023] ICES . 2008. Report of the Workshop on Small Pelagics ( *Sardina pilchardus* , *Engraulis encrasicolus* ) Maturity Stages (WKSPMAT). ICES.

[ece373792-bib-0024] INRH . 2019. Rapport Annuel de l'État des Stocks et des Pêcheries Marocaines au Maroc 2019. Département Des Pêches, Institut National de Recherche Halieutique, INRH.

[ece373792-bib-0025] Jardas, I. , M. Šantić , and A. Pallaoro . 2004. “Diet Composition and Feeding Intensity of Horse Mackerel, *Trachurus trachurus* (Osteichthyes: *Carangidae*) in the Eastern Adriatic.” Marine Biology 144: 1051–1056. 10.1007/s00227-003-1281-7.

[ece373792-bib-0026] Lema, S. C. , J. A. Luckenbach , Y. Yamamoto , and M. J. Housh . 2024. “Fish Reproduction in a Warming World: Vulnerable Points in Hormone Regulation From Sex Determination to Spawning.” Philosophical Transactions of the Royal Society, B: Biological Sciences 379, no. 1898: 20220516. 10.1098/rstb.2022.0516. PMC10838641 38310938

[ece373792-bib-0027] Murua, H. , and F. Saborido‐Rey . 2003. “Female Reproductive Strategies of Marine Fish Species of the North Atlantic.” Journal of Northwest Atlantic Fishery Science 33: 23–31. 10.2960/J.v33.a2.

[ece373792-bib-0028] Nasri, H. , S. Abdellaoui , A. Omari , et al. 2021. “Length‐Weight Relationship and Condition Factor of *Trachurus trachurus* Found in the Central‐East Region of the Moroccan Mediterranean.” Indonesian Journal of Science and Technology 6, no. 3: 457–468.

[ece373792-bib-0029] Nasri, H. , R. Sabbahi , S. Abdellaoui , et al. 2024. “Ecology, Anatomy, Reproduction, and Diet of the Atlantic Horse Mackerel, *Trachurus trachurus* : A Comprehensive Review.” Egyptian Journal of Aquatic Biology and Fisheries 28, no. 3: 517–539.

[ece373792-bib-0030] Ndjaula, H. O. , T. Hansen , M. Krüger‐Johnsen , and O. S. Kjesbu . 2009. “Oocyte Development in Captive Atlantic Horse Mackerel *Trachurus trachurus* .” ICES Journal of Marine Science 66, no. 4: 623–630. 10.1093/icesjms/fsp032.

[ece373792-bib-0031] Nunes, Y. B. S. , M. B. Aranha , J. Freitas , J. F. F. Fernandes , L. R. Silva , and M. B. Figueiredo . 2020. “Length at First Sexual Maturity of Economically Important Fishes in the Brazilian Northeast Coast.” Ocean and Coastal Research 68: e20311. 10.1590/S2675-28242020068311.

[ece373792-bib-0032] Petrik, C. M. , C. A. Stock , K. H. Andersen , P. D. Van Denderen , and J. R. Watson . 2019. “Bottom‐Up Drivers of Global Patterns of Demersal, Forage, and Pelagic Fishes.” Progress in Oceanography 176: 102124. 10.1016/j.pocean.2019.102124.

[ece373792-bib-0033] Rahmani, K. 2020. Étude de la Biologie d'un Poisson Pélagique du Genre Trachurus Dans la Baie de Béni Saf (Algerie occidentale). PhD Thesis. Djillali Liabès—Sidi Bel Abbès, Algeria.

[ece373792-bib-0034] Raya, V. , and A. Sabatés . 2015. “Diversity and Distribution of Early Life Stages of Carangid Fishes in the Northwestern Mediterranean: Responses to Environmental Drivers.” Fisheries Oceanography 24: 118–134. 10.1111/fog.12097.

[ece373792-bib-0035] Rodríguez‐Castañeda, J. C. , A. Ventero , and M. Iglesias . 2024. “Analysis of the State of Conservation of *Trachurus trachurus* in the Western Mediterranean Sea Based on the Interannual (2009–2020) Changes in Their Life History Traits.” Marine Biology 171, no. 1: 34. 10.1007/s00227-023-04356-4.

[ece373792-bib-0036] Tahari, F. Z. 2011. Contribution à l'Étude de la Biologie de la Reproduction d'un Petit Pélagique le Saurel Trachurus trachurus: Spermatogenèse, Condition, RGS, RHS. PhD Thesis. University of Oran1‐Ahmed Ben Bella, Algeria.

[ece373792-bib-0037] Taieb, A. H. , M. Ghorbel , N. B. H. Hamida , and O. Jarbaoui . 2010. “Spawning Period and Size at First Sexual Maturity of Sea Bream *Sparus aurata* in the Southern Coast of Tunisia.” In 2nd International colloquium on Biodiversity and Coastal Ecosystem, Oran, Algeria.

[ece373792-bib-0038] Van Damme, C. J. , A. Thorsen , M. Fonn , et al. 2014. “Fecundity Regulation in Horse Mackerel.” ICES Journal of Marine Science 71, no. 3: 546–558. 10.1093/icesjms/fst156.

[ece373792-bib-0039] Wahbi, F. , L. Loc'h , A. Berreho , A. Benazzouz , A. Ben Mhmed , and A. Errhif . 2015. “Composition et Variations Spatio‐Temporelles du Régime Alimentaire de *Trachurus trachurus* (*Carangidae*) de la Côte Atlantique Marocaine.” Cybium 39: 131–142.

[ece373792-bib-0040] Waldron, M. E. , and M. Kerstan . 2001. “Age Validation in Horse Mackerel ( *Trachurus trachurus* ) Otoliths.” ICES Journal of Marine Science 58: 806–813. 10.1006/jmsc.2001.1071.

[ece373792-bib-0041] Ward, A. J. W. , M. I. A. Kent , and M. M. Webster . 2020. “Social Recognition and Social Attraction in Group‐Living Fishes.” Frontiers in Ecology and Evolution 8: 15. 10.3389/fevo.2020.00015.

